# Laparoscopic versus open reduction of idiopathic intussusception in children: an updated institutional experience

**DOI:** 10.1186/s12887-022-03112-9

**Published:** 2022-01-17

**Authors:** Jian Zhao, Jun Sun, Deyu Li, Wei Jue Xu

**Affiliations:** 1 Department of Pediatric Surgery, Yangzhou Maternal and Child Health Hospital, Yangzhou, China; 2 Department of General Surgery, Shanghai Children’s Hospital, Shanghai Jiao Tong University, No.355 Luding Road, Putuo District, Shanghai, China

**Keywords:** Idiopathic intussusception, Laparoscopy, Children

## Abstract

**Background:**

In the reduction of intussusception, due to the lack of randomized, controlled, and prospective clinical trials to confirm the superiority of the laparoscopic approach over open surgery, more evidence was needed. This study aimed to compare the results of laparoscopy and open reduction of idiopathic intussusception in children as well as to illustrate some skills for the reduction of intussusception laparoscopically.

**Methods:**

A retrospective review was performed to evaluate outcomes for patients with idiopathic intussusception who were treated laparoscopically (LAP group) from January 2015 to December 2019 and to compare the outcomes with laparotomy (OPEN group) during the same period.

**Results:**

During the period studied, there were 162 patients treated surgically for intussusception: 62 LAP and 100 OPEN. No statistical differences were found in demographic data, clinical symptoms and signs, duration of symptoms, location and types of intussusception between the two groups. Conversion to open procedure was required for 11 patients in the LAP group. The operation time and time to oral intake were shorter in the LAP group while the difference was not significant. If the 11 conversion cases were excluded, the operation time and time to oral intake were significantly shorter (*P* < 0.05) in the LAP group. The length of stay was significantly shorter in the LAP group (*P* < 0.05). Intraoperative and postoperative complication rates between the two groups were comparable (*P* = 1.0).

**Conclusion:**

Laparoscopy was safe and effective in the treatment of pediatric idiopathic intussusceptions. Pediatric surgeons with sophisticated minimally invasive skills should choose laparoscopy as the first choice in the treatment of idiopathic intussusceptions.

## Introduction

Acute intussusception was one of the most common causes of acute abdominal pain in children, and it was the most frequent cause of intestinal obstruction in children aged 3 months to 5 years. The vast majority (nearly 95%) of intussusception lacked a definite pathologic lead point and were classified as idiopathic intussusceptions [1]. China was one of the countries with a high incidence of intussusception in the world [2]. The image-guided pneumatic reduction was the first-line management in our institution, and the success rate was 91.9%. Operation was indicated when pneumatic air enema was unsuccessful or who was contraindicated. Traditionally, a large right-sided transverse incision was needed for manual reduction. With the advancement of minimally invasive surgery in children, the laparoscopic approach was increasingly used in the management of intussusception [3–6]. However, no randomized, controlled, and prospective clinical trials focused on the two methods. More evidence to confirm the feasibility and safety of the laparoscopic approach in intussusception was needed.

This study aims to compare the results of laparoscopy and open reduction of idiopathic intussusception in children as well as to elaborate some skills for the management of intussusception laparoscopically.

## Methods and materials

After institutional review board approval was obtained, a retrospective chart review was conducted. Between January 2015 and December 2019, children aged < 18 years who were operated for idiopathic intussusception in our institution were reviewed. Patients were classified into two groups based on surgical methods, laparoscopy (LAP) and open surgery (OPEN). Data drawn from the patient medical records and follow-up records included demographic data (age, weight, gender), duration of symptoms, types of intussusception, location of intussusceptum, operation time, conversion, time to oral intake, length of postoperative hospital stay (LOS), complication (intraoperative, postoperative), and costs of hospitalization.

### Laparoscopic technique

**Fig. 1:**
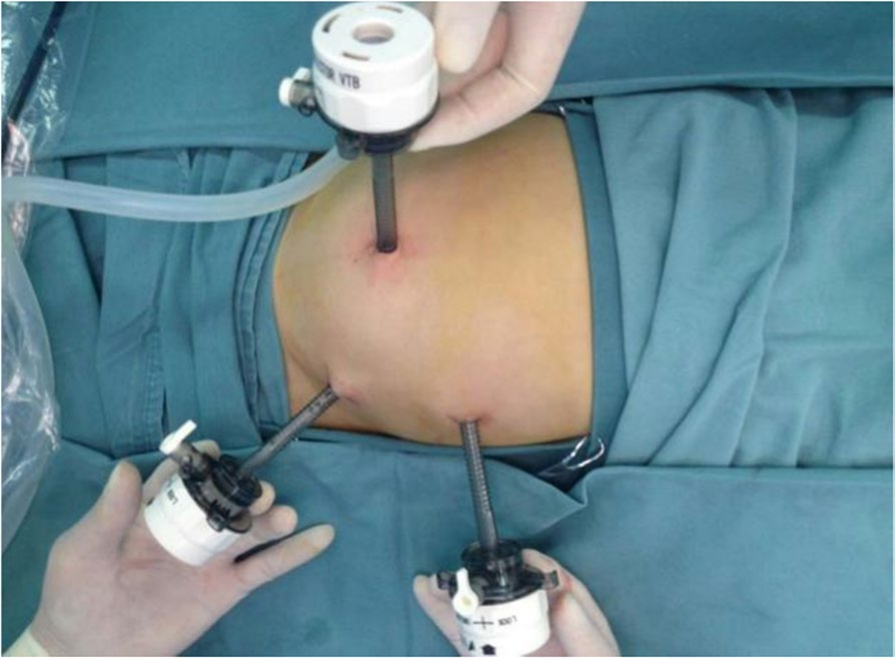
The locations of trocar placement

The patient was positioned supine. A 5 mm Trocar was inserted through the periumbilical incision using the open method. A 5 mm 30 degree laparoscopic was introduced into the peritoneal cavity. The first thing was to determine whether the intussusception was still present. The additional two 5 mm Trocar were placed in the left lower quadrant and left upper quadrant after confirmed existing intussusception (Fig. 1). If the intussusceptum was in descending colon or sigmoid colon, the additional two 5 mm Trocar were placed in the left lower quadrant and suprapubic area.

**Fig. 2:**
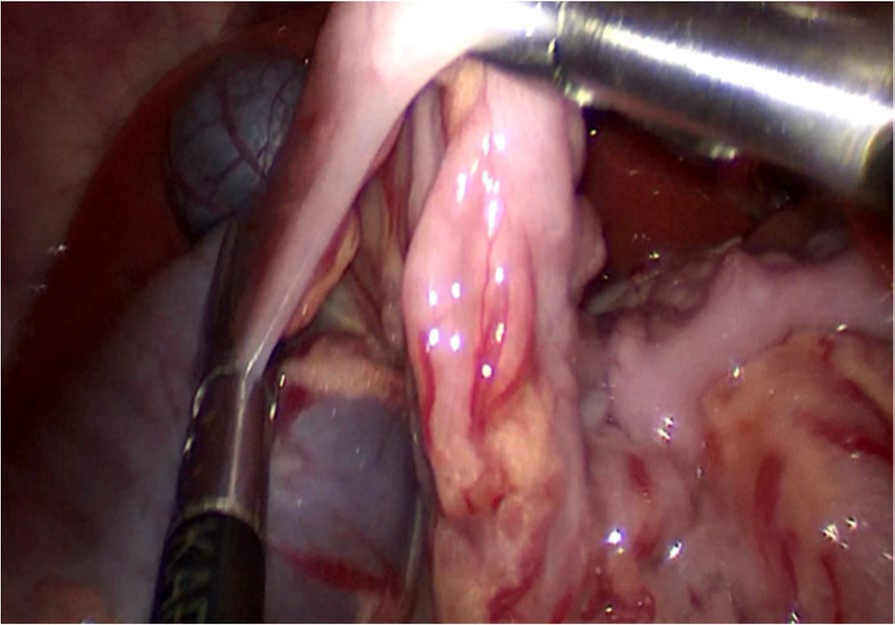
Squeeze the most distal part of the intussusceptum back towards cecum

**Fig. 3:**
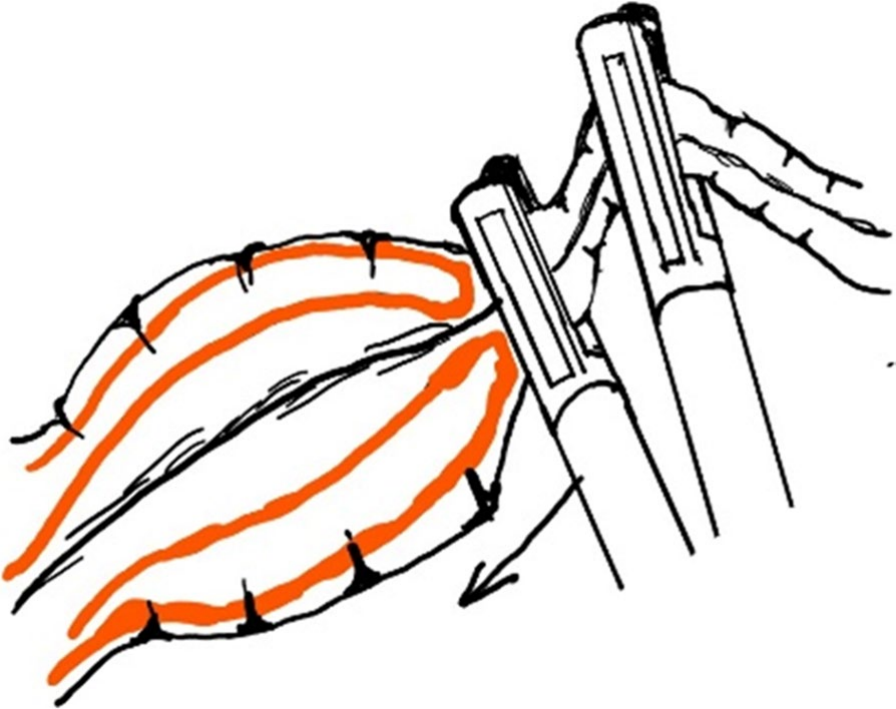
Schematic diagram of Fig. 2

**Fig. 4:**
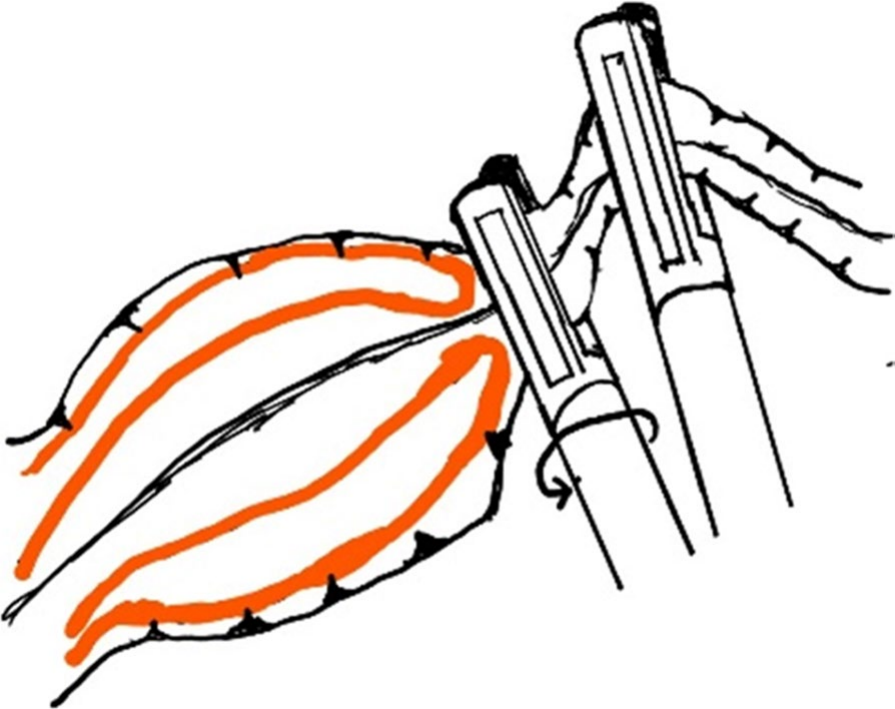
Schematic diagram of rotating the handle to press the intussusceptum forward cecum

**Fig. 5:**
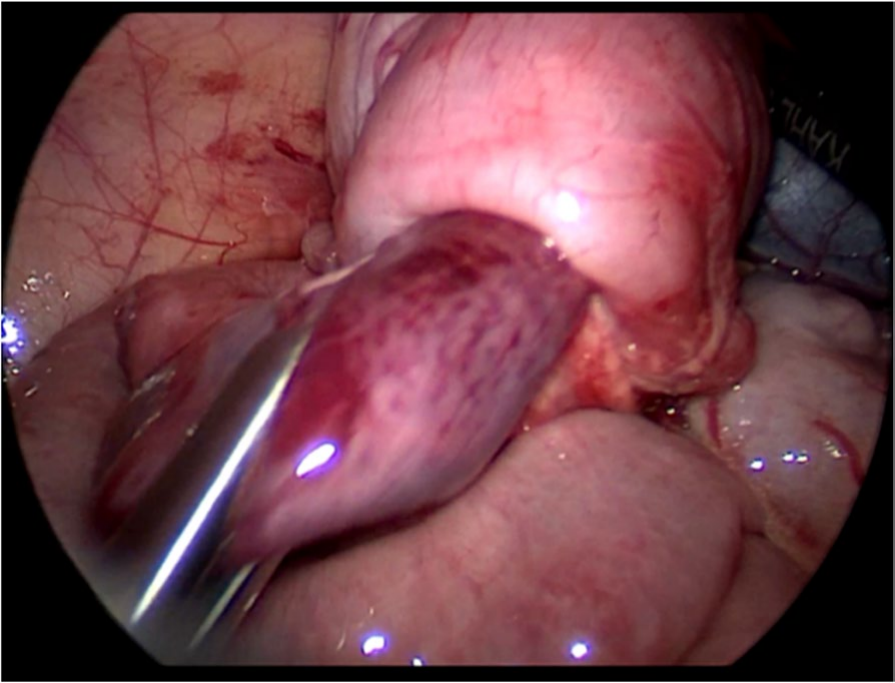
Reduce the intussusception by pulling ileum

**Fig. 6:**
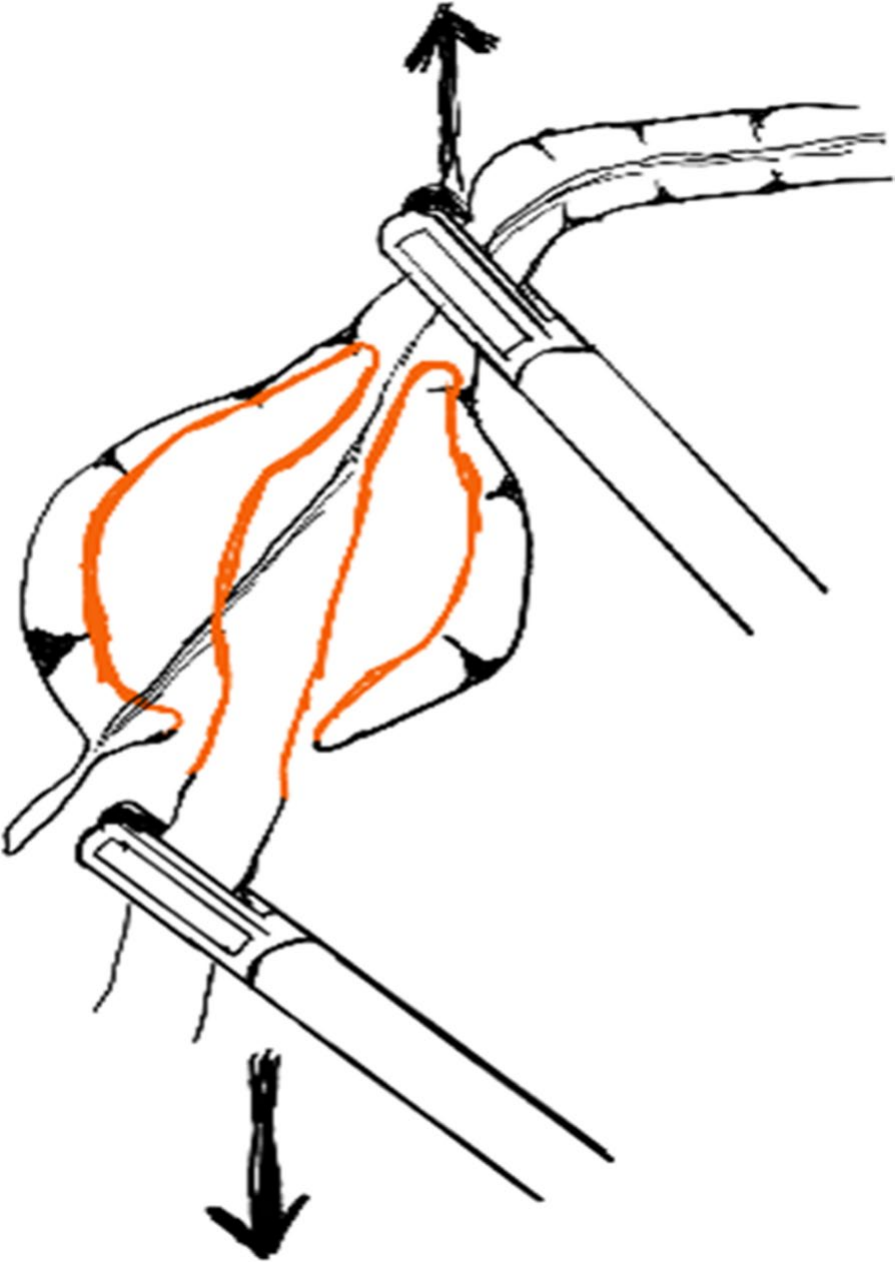
Schematic diagram of reduction of intussusception by pulling ileum

The first step was to identify the leading edge of the intussusceptum. Using two atraumatic graspers to squeeze the most distal part of the intussusceptum slowly and gently back towards cecum as much as possible (Figs. 2, 3 and 4). After that, the entire segment of the ascending colon was completely grasped and hold on as close as possible to the intussusceptum using the right hand, while using the left hand to find the small bowel and grasp the entire segment of the small intestine and applying traction to pull out the intussusceptum (Figs. 5 and 6). If the reduction was not completed, repeat the above steps several times. After reduction, careful inspection was then performed to rule out the existence of a pathologic lead point and to evaluate for any sign of ischemia, necrosis, or perforation.

### Statistics

Statistical analysis was achieved by SSPS software with χ^2^ test and Mann–Whitney U test. A 2-tailed *p* < 0.05 was chosen as the threshold for statistical significance.

## Results

**Fig. 7:**
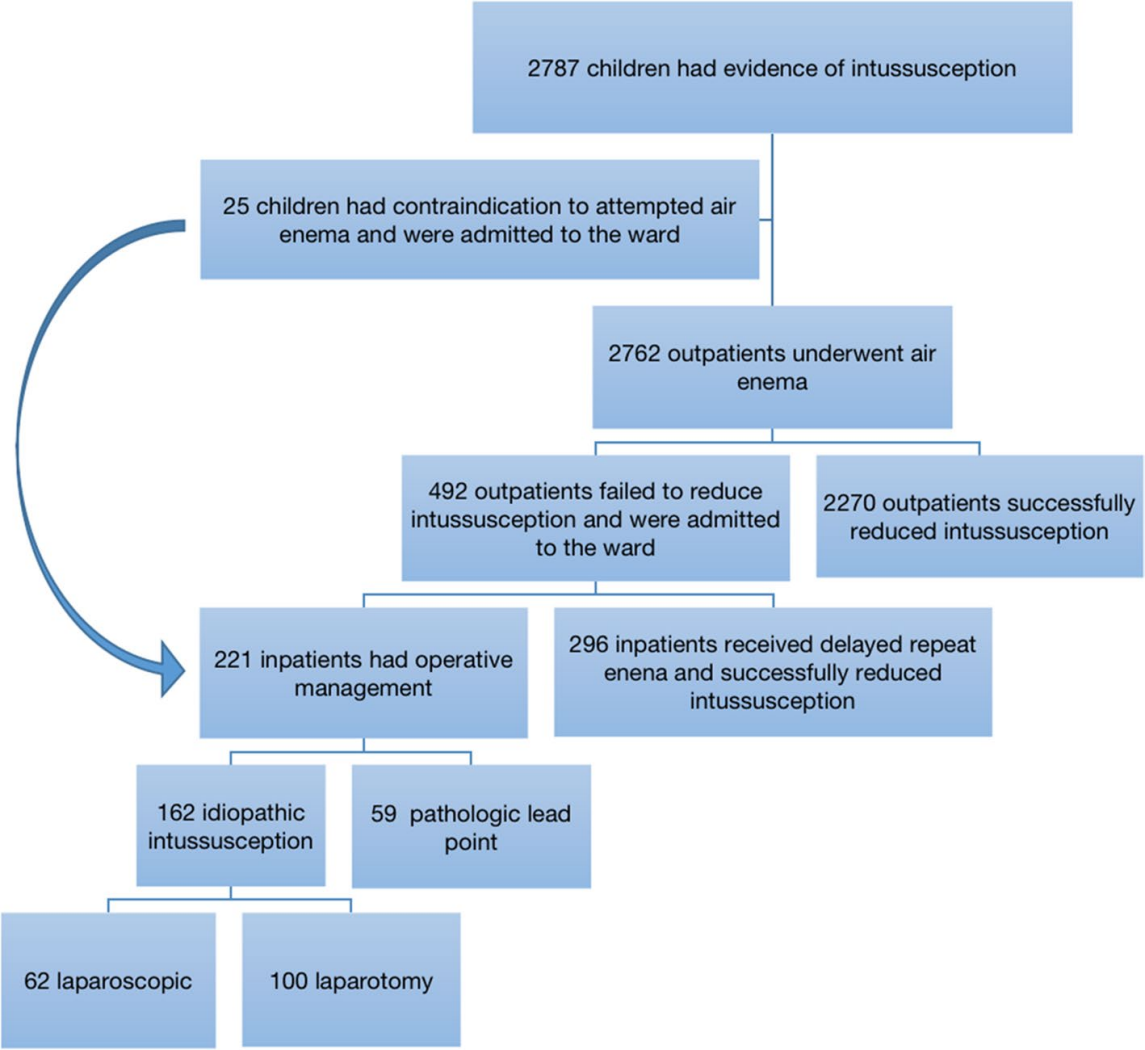
Flow chart of management results for children diagnosed with intussusception

As shown in Fig. 7, from January 1, 2015 to December 31, 2019, 2566 in 2787 children with the evidences of intussusception were released undergoing primary or repeated pneumatic reduction in our institution. During the timed period studied, 162 children required surgical intervention. Sixty two patients received laparoscopic surgery, and 100 received open surgery. Seven cases (11.3%) in the LAP group, and 9 cases (9%) in the OPEN group who underwent surgery showed no intussusception existence intraoperatively. The conversion was required in 11 patients in the LAP group. Eight patients were failed laparoscopic reduction because of ‘tight’ intussusception. Two patients were unable to exclude a pathological lead point after laparoscopic success reduction. One patient was iatrogenic ileal perforation and failed laparoscopic reduction.

The comparison of patient’s characteristics was shown in Table 1. Between the OPEN and the LAP groups, no statistical differences were found in gender, age, weight, clinical symptoms and signs (abdominal pain, vomiting, rectal bleeding, abdominal mass, fever), duration of symptoms, location and types of intussusception.

**Table 1:** The characteristics of intussusception patients receiving operation

	LAP (*N* = 62)	OPEN (*N* = 100)	*P*-value
Gender (Male / Female)	42 / 20	72 / 28	0.598
Age (months)	10.0 (2, 66)	9.0 (1, 69)	0.130
Weight (kg)	10.0 (6.6, 26.0)	10.0 (5.4, 22.5)	0.255
Length of symptoms (hours)	17.0 (1.0, 96)	15.5 (4.0, 96.0)	0.557
Clinical symptoms and signs			
Abdominal pain	54 (87.1%)	80 (80%)	0.290
Vomiting	44 (70.9%)	81 (81%)	0.178
Rectal bleeding	40 (64.5%)	76 (76%)	0.151
Abdominal mass	41 (66.1%)	61 (61%)	0.616
Fever	8 (12.9%)	17 (17%)	0.655
Leading edge of the intussusceptum			
Ascending (include hepatic flexure)	32 (53.2%)	59 (59%)	0.416
Transverse (include splenic flexure)	16 (25.8%)	26 (26%)	1.000
Descending	3 (4.84%)	3 (3%)	0.676
Sigmoid	3 (4.84%)	1 (1%)	0.157
Ileum	1 (1.61%)	2 (2%)	1.000
Spontaneous reduction	7 (11.3%)	9 (9%)	0.787
Type of intussusception			
Ileocolic	52 (83.9%)	82 (82%)	0.833
Ileoileocolic	2 (3.2%)	7 (7%)	0.484
Ileoileal	1 (1.6%)	2 (2%)	1.000
Spontaneous reduction	7 (11.3%)	9 (9%)	0.787

The operation time in the LAP group (mean, 58, 24 to 184) was shorter than that in the OPEN group (mean 59, 30 to 265), but differences between the two groups were not statistically significant, *P* = 0.45. If eleven conversion cases were excluded, the operation time in the LAP group (mean, 45, 24 to 145) was statistically significantly shorter than that in the OPEN group (mean 59, 30 to 265), *P* = 0.008. The time to oral intake (day) in the LAP group (mean, 2, 1 to 6) was shorter than that in the OPEN group (mean 2, 1 to 7), but differences were not statistically significant, *P* = 0.219. If the conversion cases were excluded, the time to oral intake in the LAP group (mean, 2, 1 to 6) was statistically significantly shorter than that in the OPEN group (mean 2, 1 to 7), *P* = 0.042. The length of stay in the LAP group was statistically significantly shorter than that in the OPEN group, *P* < 0.05. The overall hospital costs were statistically significantly increased in the LAP group over the OPEN group, *P* = 0.000. As is shown in Table 2.

**Table 2:** Main outcome between the LAP and the OPEN group

	LAP	OPEN (*N* = 100)	*P*-value
Operation time (min) N = 62	58 (24–184)	59 (30–265)	0.45
Conversion excluded *N* = 51	45 (24–145)		0.008*
Time to oral intake (day)	2.0 (1.0–6.0)	2.0 (1.0–7.0)	0.219
Conversion excluded	2.0 (1.0–6.0)		0.042*
Length of stay (day)	5.0 (3.0–8.0)	5.0 (3.0–12.0)	0.042*
Conversion excluded	4.0 (3.0–8.0)		0.009*
Total costs (CNY)	15,778 (11197–33,930)	12,638 (6760–42,480)	0.000*
Conversion excluded	15,476 (11197–33,930)		0.000*
No. of bowel resections	4	10	0.570
Conversion excluded	0		0.017*
No. of intraoperative complications	12 (19.4%)	13 (13%)	0.371
Conversion excluded	5 (9.8%)		0.791
No. of postoperative complications	5 (8.1%)	8 (8%)	1.000
Conversion excluded	4 (7.8%)		1.000

Intraoperative complications in the LAP group were noted in twelve cases (19.4%) included nine colonic serosa tearing, two ileum serosa tearing, and one iatrogenic ileal perforation. In the OPEN group that were noted in thirteen cases (13%) included seven colonic serosa tearing, four ileum serosa tearing, and two iatrogenic ascending colon perforation. There were no significant differences between the two groups, *P* = 0.371.

The median follow-up was 34 months (range, 4 to 63 months). Postoperative complications in the LAP group were noted in five cases (8.1%) included three recurrences, two Trocar-site hernia. In the OPEN group that were noted in eight cases (8%) included five recurrences, one small bowel obstruction, one wound infection, and one pelvic abscess. There were no significant differences between the two groups, *P* = 1.000.

## Discussion

Air reduction of intussusception was the first-line management and the overall success rate was 92.1% (2566 cases in 2787 cases) in our institution. Nevertheless, in cases of failed pneumatic enema reduction or existed contraindications for pneumatic reduction, surgical treatment remained indispensable. Traditionally, a large right-sided transverse incision was needed for manual reduction. In 1996, Cuckow et al. [7] reported, for the first time, laparoscopic reduction of idiopathic intussusception in a 10-month-old boy. Since then, laparoscopy had been increasingly used in pediatric intussusception surgery. Our institution had started laparoscopic intussusception reduction since 2012, and the current success rate elevated to more than 85%.

Although there were some articles about the laparoscopic reduction of intussusception, the detailed steps for laparoscopic reduction of intussusception were rarely described. Our experience was, using two atraumatic graspers to grasp the entire segment of the colon and squeeze the most distal part of the intussusceptum back towards cecum (Figs. 2 and 3). If the resistance was significant, you can rotate the handle to press the intussusceptum forward cecum (Fig. 4). When the intussusceptum reach ascending colon, the entire segment of the ascending colon was completely grasped and hold on as close as possible to the intussusceptum using the right hand, using the left hand to find the small bowel and grasp the entire segment of the small intestine and applying traction to pull out the intussusceptum firmly and gently (Figs. 5 and 6). Remember, grasp only small portions of the bowel wall might result in disastrous tears of the bowel wall. Afterwards, carefully inspection was performed to rule out the existence of a pathologic lead point and to evaluate for any sign of ischemia, necrosis, or perforation.

In this series, the conversion rate from LAP to OPEN was 17.7%. The most common conversion reason was unable to reduce the intussusception. Looking at the leading edge of the intussusceptum, we found that of the 11 conversion cases, 5 cases (45.5%) of intussusceptum were located distal to the splenic flexure colon, which was significantly higher than that of conversion excluded LAP group (*P* < 0.05). It seems a long segment of intussusception was positively related to conversion.

Spontaneous reduction of ileocolic intussusception after unsuccessful pneumatic reduction had been reported in approximately 10% of cases [4, 8]. In this study, 16 patients (9.89%) were found with negative intraoperative evidence of intussusception after an unsuccessful pneumatic enema reduction. If these patients choose laparoscopic surgery, it can be found to avoid unnecessary surgical trauma in time (only 1 or 2 5 mm incisions).

With the advancement of minimally invasive surgery in children, the laparoscopic approach was increasingly used in the management of intussusception. Many scholars reported that laparoscopic surgery could reduce the time of postoperative fasting and hospital stay, and did not increase the operation time [6, 8–12]. This study revealed that the operation time and the time to oral intake in the LAP group was shorter than in the OPEN group. If the conversion cases were excluded, the differences between the two groups were statistically different. The length of stay in the LAP group was statistically significantly shorter than that in the OPEN group. These results suggested the superiority of laparoscopic over open surgery. Although the overall hospital costs were increased in the LAP group, the time to oral intake and length of stay in the LAP group were shorter, which narrow the cost gap.

As showed in Table 2, the incidence of intraoperative and postoperative complications was similar between the two groups. The most common intraoperative complication in both groups was serosa tearing. There was one iatrogenic ileal perforation in the laparoscopic group. Our traditional concept was that in the reduction of idiopathic intussusception, we should squeeze the colon and should not pull the ileum. So iatrogenic small intestine perforation rarely occured in open surgery. In laparoscopic surgery, it was almost impossible to reduce intussusception by merely squeezing the colon, and it was essential to pull the ileum to reduce the intussusception. If the surgeon grasped only small portions of the bowel wall or pulls too violently, it might result in disastrous tears of the bowel wall. Our lesson was to grasp the entire segment of the bowel and applying traction firmly and gently. The surgeon should have sophisticated minimally invasive skills, so as to detect the tight tear in the serosa timely. The most common postoperative complication was intussusception recurrence. In our center, the recurrent rate was 4.9%. Yet, it was reported that the recurrence rate after successful enema reduction as high as 20% with an average of about 10% [13]. The etiology of the difference was still unclear. There were two Trocar-site hernias in the laparoscopic group. One case was in the left upper quadrant, and the content was omentum. When the Trocar was pulled out, the omentum was brought into the wound. The other was in the left lower quadrant, and the content was the wall of the sigmoid colon, forming a Richter’s hernia. Our lesson was to remove the Trocar under the direct view after laparoscopic surgery, and 5-mm fascial incisions were needed to be closed in the toddler.

Meanwhile, several limitations were existed in our study. First, it was a single-center retrospective study, not a randomized, controlled, and prospective clinical trial. Second, there was a potential for selection bias. The patient’s symptoms and signs and the surgeon’s personal preferences would affect the choice of surgical method. Finally, the sample size was not large enough.

In conclusion, laparoscopy was a safe and effective method in the treatment of pediatric idiopathic intussusceptions. Pediatric surgeons with sophisticated minimally invasive skills could choose laparoscopy as the first choice in the treatment of idiopathic intussusceptions,.

## Data Availability

The datasets used or analysed during the current study are available from the corresponding author on reasonable request.

## Abbreviations

LAP: Laparoscopy
OPEN: Open surgery
LOS: Length of postoperative hospital stay
